# Opsoclonus-Myoclonus Syndrome Associated With West-Nile Virus Infection: Case Report and Review of the Literature

**DOI:** 10.3389/fneur.2018.00864

**Published:** 2018-10-16

**Authors:** Răzvan Alexandru Radu, Elena Oana Terecoasă, Amalia Ene, Ovidiu Alexandru Băjenaru, Cristina Tiu

**Affiliations:** ^1^Department of Neurology, University Emergency Hospital Bucharest, Bucharest, Romania; ^2^Department of Clinical Neurosciences, University of Medicine and Pharmacy Carol Davila, Bucharest, Romania

**Keywords:** opsoclonus-myoclonus syndrome, West-Nile virus, West-Nile encephalitis, outcome, treatment

## Abstract

Opsoclonus-myoclonus syndrome (OMS) is a very rare condition with different autoimmune, infectious and paraneoplastic aetiologies or in most cases idiopathic. We report the case of a 75-year-old woman who was admitted in our department in early fall for altered mental status, opsoclonus, multifocal myoclonus, truncal titubation and generalized tremor, preceded by a 5 day prodrome consisting of malaise, nausea, fever and vomiting. Brain computed tomography and MRI scans showed no significant abnormalities and cerebrospinal fluid changes consisted of mildly increased protein content and number of white cells. Work-up for paraneoplastic and autoimmune causes of OMS was negative but serologic tests identified positive IgM and IgG antibodies against West Nile virus (WNV). The patient was treated with Dexamethasone and Clonazepam with progressive improvement of mental status, myoclonus, opsoclonus and associated neurologic signs. Six months after the acute illness she had complete recovery. To our knowledge this is the 14th case of WNV associated OMS reported in the literature so far. We briefly describe the clinical course of the other reported cases together with the different treatment strategies that have been employed.

## Introduction

Opsoclonus-myoclonus syndrome (OMS) is a very rare condition characterized by erratic multidirectional eye movements without intersaccadic intervals accompanied by myoclonus affecting both the limbs and the trunk, cerebellar ataxia, tremor and encephalopathy (1). Most cases of OMS in adults are paraneoplastic or idiopathic (2, 3) but the syndrome may also occur in association with a wide spectrum of infections with different agents including several viruses (influenza, varicella, human herpes virus 6, HIV, enteroviruses, Epstein–Barr, Cytomegalovirus, Coxsackie, West Nile Virus), Borrelia, Salmonella, Mycoplasma pneumoniae or as a post-streptococcal syndrome (4). Although West Nile virus (WNV) is listed among infectious causes of OMS in almost all publications related to this topic, through a comprehensive electronic search for English language publications performed in April 2018 using Pubmed, Medline and Web of Science databases we identified only 13 reported cases of OMS associated with WNV infection.

We report a case of OMS associated with WNV encephalitis and review the clinical characteristics, outcome and treatment options of the other reported cases.

## Case report

A 75-year-old Caucasian woman was admitted in our department in early fall for altered mental status, opsoclonus, multifocal myoclonus, truncal titubation, and generalized tremor. She had been in usual health until approximately 5 days before, when she was admitted to an internal medicine ward for malaise, tachypnea, nausea, vomiting, and fever. At that moment the symptoms were considered to be suggestive for an upper respiratory tract infection and she was treated with antipyretics. During the fourth day of hospitalization the patient gradually became confused and developed involuntary muscle twitches of all limbs, tremor and “jerky eye movements” which prompted evaluation by a neurologist. Her past medical history consisted of arterial hypertension, diabetes mellitus and chronic autoimmune thyroiditis.

At admission in our department, the patient was somnolent, afebrile, not oriented to person, place and time and was only able to answer “yes” or “no” to simple questions. The pulse was 80 beats per minute, the blood pressure 150/90 mmHg, the respiratory rate 21 breaths per minute and the oxygen saturation 96% while breathing ambient air. Neurological examination revealed mild nuchal rigidity, opsoclonus, bilateral and multifocal myoclonus, more severely affecting the arms than the legs, truncal titubation amplified during active movements, postural and intention tremor, bilateral palmomental reflex, and mild weakness of the right upper limb. She could not maintain a sitting position due to truncal titubation and severe myoclonus.

Initial work-up done in the Department of Internal Medicine was unremarkable with the exception of a mild inflammatory syndrome and mildly increased creatinine kinase levels. Brain computed tomography (CT) scan performed at admission in our department showed leukoaraiosis, without other significant changes. Lumbar puncture revealed normal opening pressure and the cerebrospinal fluid analysis identified 17 leukocytes/mm^3^, mildly elevated albumin levels (30.6mg/l, local reference range 5–20 mg/l) and normal glucose levels. The electroencephalogram showed slow bilateral frontal waves. At this point we established a presumptive diagnosis of rhombencephalitis with opsoclonus-myoclonus syndrome (OMS) and started work-up for likely causes. Contrast enhanced brain MRI performed 10 days after symptom onset identified bilateral relatively symmetrical scattered white matter hyperintensities, predominantly in the periventricular and subcortical regions, consistent with small vessel disease related changes and no other significant abnormalities. A CT scan of the chest, abdomen and pelvis was performed in order to search for a possible tumor. Results were normal with the exception of a small non-enhancing hypodense lesion in the right ovary which was not correlated with raised CA-125 levels and was considered by the gynecologist an incidental finding. Further work-up for infectious, paraneoplastic, and autoimmune causes of encephalitis (see Table 1) was unremarkable, except for positive IgM and IgG antibodies against WNV.

**Table 1:** Laboratory work-up.

	**Result**	**Reference range**
**INFECTIOUS DISEASES SEROLOGY (SERUM TESTING)**		
Epstein-Barr Virus antibodies IgG	594 U/ml	Negative <20 U/ml
Epstein-Barr Virus antibodies IgM	<10 U/ml	Negative <20 U/ml
Mycoplasma pneumoniae antibodies IgG	<9 U/ml	Negative <9 U/ml
Mycoplasma pneumoniae antibodies IgM	<0.9 U/ml	Negative <0.9 U/ml
Mycoplasma pneumoniae antibodies IgA	<9 U/ml	Negative <9 U/ml
Listeria Monocytogenes IgM/IgG	Negative	Negative
West-Nile antibodies IgG^1^	2.58	< 1.1 (index)
West-Nile antibodies IgM^1^	4.16	< 1.1 (index)
West-Nile antibodies IgG^2^	3.25	< 1.1 (index)
West-Nile antibodies IgM^2^	3.41	< 1.1 (index)
**ANTI-NEURONAL ANTIBODIES**		
anti-Amphiphysin, anti-CV2, anti-PNMA2, anti-Ri, anti-Yo, anti-Hu, anti-Recoverin, anti-Sox1, anti-Titin	Negative	Negative (Immunoblot)
**TUMOR MARKERS**		
CEA	5.4 ng/ml	0 – 5.8 ng/ml
α-fetoprotein	5.8 ng/ml	0 – 9.5 ng/ml
Neuron – specific enolase	9.95 ng/ml	< 17 ng/ml
CA 19-9	26.6 U/ml	0 – 37 U/ml
CA 15-3	21.5 U/ml	0 – 23 U/ml
CA 125	21.7 U/ml	0 – 35 U/ml
**OTHERS**		
Anti-voltage gated K channels antibodies	Negative	Negative
NMDA receptor antibodies	Negative	Negative
Glutamic Acid Decarboxylase-II antibodies	<5 IE/ml	< 10 IE/ml

1 Blood samples collected 8 days after clinical onset;

2 *blood samples collected 14 days after first test*.

Upon transfer in our department the patient was immediately given empiric therapy with Ampicillin, Ceftriaxone, Vancomycin, and Dexamethasone to cover potential causes of encephalitis and Clonazepam as a symptomatic therapy for myoclonus. The antibiotics were discontinued after the results of the lumbar puncture. Within a couple of days, myoclonus showed visible improvement but the opsoclonus and the cognitive status remained unchanged. Two weeks later, the patient started to improve from the cognitive point of view, as she was more aware of her surroundings and became oriented in time and partly in space. A second electroencephalogram performed at this point showed normal cerebral activity. She was discharged 3 weeks after admission, being able to walk with unilateral support. Neurocognitive evaluation performed at discharge showed severe visuospatial deficit and cognitive impairment (Mini Mental Status score—MMSE—of 12 points, Clock drawing test 2/10 points).

During the following weeks her condition gradually improved and she began to perform more and more activities of daily living by herself. At the follow-up visit, performed 1 month after discharge, neurological examination was normal except for very mild action tremor and mild opsoclonus which was only evident at close examination. Her neurocognitive status greatly improved, as she had a MMSE score of 25 points. A second follow-up visit, performed 6 months after discharge, showed normal neurological examination with complete resolution of previous signs and symptoms. Neurocognitive evaluation was within accepted ranges for her age and intellectual background (MMSE 29 points, Clock drawing test 10/10 points).

## Discussion

### Epidemiology of west-nile virus

WNV is a member of the Japanese encephalitis antigenic sero-complex, belonging to the family of Flaviviridae, which also includes Japanese encephalitis virus, St Louis encephalitis virus, Dengue Virus, Yellow Fever Virus and the emerging Zika Virus (5, 6). It was first identified in the West Nile province of Uganda in 1937 (7). Since the initial communication, several WNV outbreaks were reported in Africa and in the Middle East, but significant disease activity was identified in Europe and North-America only after 1990 (8). The first major outbreak in Europe was described in south-east Romania in 1996 (9). Nowadays, cases of WNV infection are reported in many European countries, but the incidence is higher in the central and southern regions (10).

In nature, WNV is maintained in a bird-mosquito-bird transmission cycle. Birds develop sustained viremia and are considered amplifier hosts while humans usually develop transient low-level serum viremia and are unlikely to act as a reservoir for the virus. Transmission to humans commonly occurs through the Culex Mosquito spp. but transmission by blood transfusion, organ donation, pregnancy, and lactation have also been identified or postulated (5).

WNV infections usually start in summer and peak in early fall due to the natural life cycle of the mosquitos (9, 11). Local risk factors that might contribute to WNV amplification in the environment include elevated ambient temperature, heavy rains, stagnant water sources and immunologically naive avian host reservoirs (12). The onset of symptoms of our patient and of two other reported cases of OMS associated with WNV infection was in the typical early fall period. Although there are no reported details about the season of disease onset for the other similar published cases, WNV infection should probably be highly considered and searched for in all cases of OMS of undetermined etiology with onset during early fall.

### West nile virus infection and symptoms

Up to 70% of the individuals infected with WNV will remain asymptomatic or will develop only minor, non-specific symptoms while one third will present with West Nile fever or other rare forms of the disease (13, 14). The clinical picture of West Nile fever is indistinguishable from other flu-like illnesses and includes: fever, headache, malaise, myalgia, nausea, vomiting, abdominal pain, and a morbilliform or maculopapular rash (13, 15). Although symptoms are usually self-limited, some patients develop persistent complaints such as fatigue, memory impairment or headache (16, 17).

Neuroinvasive disease occurs in <1% of patients who develop clinical manifestations of WNV infection and can manifest as one of the following three clinical entities: WNV meningitis, WNV encephalitis or acute flaccid paralysis (18). Established risk factors for neuroinvasive disease include: advanced age, history of cardiovascular disease, chronic kidney disease, hepatitis C infection, immunosuppression, and particular genomic determinants involving adaptive and innate immune response pathways (12).

Neuroinvasive mechanisms for WNV are not yet clarified with both haematogenous and trans-neural pathways being proposed (19). The most likely hypothesis suggests a trans-endothelial route of entry whereby the virus is trafficked through an altered blood brain barrier by infected leucokytes (20, 21). The different clinical phenotypes reported in neuroinvasive WNV infection might be explained by the innate differences in antiviral cytokines and interferon stimulated-genes expressed in different neural subtypes (22). Recent studies on experimental animal models identified a mutation in the interferon stimulated-gene Ifi27l2a which promotes location specific neuronal cell death. In Ifi27l2a^−/−^ and WNV infected mice, neurons in the cerebellum, brainstem, and possibly spinal cord live longer and harbor higher WNV burden in the active phase of the disease (23).

The acute development of a rash during the initial phase of the disease is thought to be significantly more rare in patients who further develop WNV encephalitis (24), but the mechanisms underlying this finding are yet unknown. However, two of the reported cases of OMS associated with WNV infection developed rash over the neck and trunk during the early course of the disease. Our patient did not show any skin changes prior to or during hospitalization.

Patients with West Nile encephalitis most commonly present with altered mental status of different severity, ranging from mild confusion to severe encephalopathy and coma. Extrapyramidal disorders are commonly observed, myoclonus, and tremor being the most frequently described (11, 25, 26). Ataxia and various ocular motility disorders, including nystagmus and opsoclonus are also consistently reported (27–30). Although West Nile infection was reported as a cause of rhombencephalitis (31), only 13 cases of OMS were described so far in the literature (see Table 2), the first case being published in 2004 (11). However, since many authors report cases of West Nile virus encephalitis in which myoclonus is accompanied by abnormal nystagmoid eye movements, the true incidence of OMS in WNV infection is probably underestimated (11, 27, 40).

**Table 2:** Clinical, imaging and laboratory characteristics of WNV associated OMS patients.

**Case**	**Age**	**Sex**	**Comorbidities**	**Prodrome & duration (days)**		**Signs and Symptoms**	**CSF**	**Treatment**	**MRI & EEG**	**Outcome**
Sayao et al. (11)	39	F	-	Headache, Nausea Vomiting Fever Imbalance	7 days	Opsoclonus, mild left facial palsy, bilateral mild intention tremor of the upper extremities, left arm myoclonus	-	Supportive care	Brain MRI: normal	Resolution of OMS Persistence of mild ataxia
Afzal et al. (32)	43	F	-	Dizziness Nausea Fever Myalgia	10 days	Opsoclonus, myoclonus, gait ataxia	92 WBC Proteins 110 mg/dl Normal glucose	IVIG i.v and oral steroids Supportive care	Brain MRI: normal	Resolution of OMS after ten days Near complete recovery
Birlutiu et al. (33)	57	F	Larynx neoplasm (4 years before) Diabetes mellitus	Dizziness Fever Vomiting Diarrhea Sleepiness	2 days	Opsoclonus (persisting during sleep), myoclonus, neck stiffness, ataxia	49 WBC Proteins 42mg/dl Normal glucose	IV Dexamethasone Clonazepam Supportive care	Brain MRI: SVD	Died 4 weeks after admission
Cooper et al. (4)	48	F	Endometrial cancer (with previous chemotherapy)	Confusion Nuchal rigidity	7 days	Opsoclonus, myoclonus mainly in the lower extremities, later coma	67 WBC Proteins 131mg/dl Normal glucose	Plasmapheresis Supportive care	Brain MRI: normal EEG: severe diffuse slowing	Slow resolution of OMS Complete neurologic rehabilitation after 90 days
Hébert et al. (34)	63	F	-	Headache Malaise Nausea Fever Gait ataxia	5 days	Opsoclonus, Jaw tremor, Mild bilateral upper limb tremor, Multifocal myoclonus	36 WBC Proteins 48mg/dl Normal glucose	IVIG Supportive care	Brain MRI: normal	Rapid resolution of myoclonus Near complete resolution of opsoclonus after 3 days Complete remission at two months
Khosla et al. (30)	62	M	Stage IV non-small cell lung cancer	Fever Lethargy	10 days	Neck stiffness Encephalopathy Truncal-limb ataxia Myoclonus Opsoclonus	17 WBC Albumin 30.6 mg/dl Normal glucose	Supportive care	Brain MRI: normal	Resolution of OMS after 60 days Persistence of ataxia Improved mental status
Alshekhlee et al. (35)	53	M	Hypertension Coronary artery disease Peripheral artery disease	Malaise Irritability Fever Headache Cervical pain	few days	Neck stiffness, encephalopathy, myoclonus, opsoclonus, intermittent action tremor	81 WBC Proteins 110 g/dl Normal glucose	-	EEG: mild slowing of the posterior dominant rhythm	Severe disability after three months Severe myoclonus and encephalopathy Improved after 8 months, showing only mild action tremor
Rasmussen et al. (36) (poster)	52	M	-	Fever Rash Headache Back-pain	-	Myoclonus, Opsoclonus Poor vision	↑ WBC ↑ protein ↑IgGIndex	Steroids and IgG Rituximab Supportive care	-	Improvement of opsoclonus Persisting myoclonus at discharge
Shellenback et al. (37) (poster)	69	M	Liver transplant (6 years before) Tacrolimus therapy	Fatigue Fever Nausea Diarrhea	14 days	Myoclonus, Opsoclonus, Coma	↑ WBC ↑ protein WNV-Ab	Supportive care	Brain MRI: thalamic T2W hyperintensity EEG: diffuse slowing	Discharged from intensive care unit No additional data
Tan et al. (38). (poster)	47	M	-	Malaise Rash Fever Headache Arthralgia	10 days	Opsoclonus, Myoclonus, Generalized ataxia	46 WBC Proteins 105mg/dl	IV Methylprednisolone 1g/5 days	Brain MRI: normal	Resolution of OMS Persistence of mild ataxia
	47	F	-	Rash Fever Arthralgia Headache	7 days	Opsoclonus, Myoclonus, Ataxic gait	76 WBC Proteins 78mg/dl	IV Methylprednisolone 1g/ 5 days	Brain MRI: normal	Resolution of OMS after 30 days Complete recovery
Zaltzman et al. (39)	61	F	Diabetes Mellitus Obesity Hyperlipidemia Hypertension	Fever Nausea Vomiting Dizziness	14 days	Opsoclonus, Myoclonus, Tremor of the head, Truncal ataxia	13 WBC	IV Methylprednisolone 1g/ 5 days	Brain MRI: SVD	Resolution of OMS after 90 days Complete recovery
	55	M	-	Fever General Weakness Vomiting Dizziness	14 days	Myoclonus, Opsoclonus and ocular, flutter, Truncal ataxia, Action tremor	-	-	Brain MRI: SVD	Complete recovery after several months
Our patient	75	F	Hypertension Diabetes mellitus Autoimmune thyroiditis	Headache Nausea Vomiting Fever	5 days	Neck stiffness, Opsoclonus, Myoclonus, Encephalopathy	17 WBC Albumin 30.6mg/dl Normal glucose	IV Dexamethasone Supportive care	Brain MRI: SVD EEG: diffuse slowing	Slow improvement Resolution of myoclonus after 21 days Resolution of opsoclonus after 60 days Complete recovery after 6 months

*EEG, Electroencephalography; MRI, Magnetic Resonance Imaging; SVD, Small Vessel Disease; WBC, White Blood Cells; M, Male; F, Female; WNV-Ab, antibodies against West-Nile Virus; IVIG, intravenous immunoglobulin; OMS, opsoclonus – myoclonus syndrome*.

### Opsoclonus-myoclonus syndrome

OMS is a rare disease in adults. A literature search regarding adult-onset OMS performed in 2012 identified <150 reported cases over a nearly 50-year period.(2) When the syndrome is not considered idiopathic, the most frequent identified etiologies are either paraneoplastic, or para-infectious, but the exact mechanisms of the disease and the neural structures involved are still not completely elucidated, due to the small number of cases and the absence of comprehensive pre-clinical and clinical studies. Several hypotheses regarding the affected neurons and neurotransmitters in this disease have been proposed. The first hypothesis postulates that alterations in the membrane properties of the saccadic burst neurons reduce the efficacy of omnipause neurons in the nucleus raphe interpositus of the pons either through excessive postinhibitory rebound or through a malfunction of the glycine receptors. However, to date, no autopsy study showed any histological changes of the omnipause cells. The second hypothesis states that the malfunction of the Purkinje and granular cells in the dorsal vermis might lead to deafferentation of the fastigial nucleus and further on to a reduced inhibitory effect of the omnipause neurons, rendering saccadic neurons free to oscillate (41, 42). Several PET and fMRI studies have confirmed the hypermetabolism of the fastigial area in OMS patients, supporting this theory (43, 44). However, in WNV associated OMS the neuronal dysfunction probably extends to more widespread areas including the brainstem and the cerebellum, thus causing abnormally occurring discharges of saccadic generators in the lower pons. Furthermore, the acute dysfunction of the cerebellum could lead to a phenomenon of cerebello-cortical diaschisis which might explain the associated encephalopathy and the cognitive sequelae seen in some OMS patients (45, 46).

The main characteristics of all reported cases of WNV associated OMS are summarized in Table 2. Three of the patients had a history of cancer and underwent prior chemotherapy, one suffered liver transplantation 6 years before and received immunosuppressive treatment. Three other patients, all aged more than 50 years, had a history of arterial hypertension and two of them also had diabetes mellitus. With the exception of one case with a very severe and rapidly progressing clinical course, all other patients developed symptoms suggestive of West Nile fever 5 to 14 days before the occurrence of neurological signs. Altered mental status, which was not predictive of unfavorable outcome, was reported in 4 patients and usually developed after hospital admission. Of the two patients with deep coma, one made a full recovery whereas details about the long-term outcome of the other patient were not available.

Since the majority of reported patients with WNV associated OMS showed no supratentorial lesions on MRI, altered mental status and EEG changes could be interpreted either in the context of a diffuse gray matter dysfunction induced by the viral infection, or in the context of a secondary cerebello-cortical network impairment. Previous research on this topic has shown that focal brainstem lesions can cause widespread cortical hypoperfusion with secondary dysfunction (45).

Details about brain MRI findings performed during the acute phase of the disease were available for 11 of the 13 reported cases of WNV associated OMS. No imaging findings consistent with brainstem involvement were identified. The most frequent reported findings were stigmata of small vessel disease in older individuals. Details about imaging findings on follow-up brain MRI scans performed weeks or months after the acute illness were not available. However, a recent small study comparing brain volumetry of healthy controls and patients with previous ocular flutter or opsoclonus showed a decrease of the cerebellar cortex volume with preservation of the cerebellar white matter volume and a subsequent decrease in both cortical and subcortical gray matter of the cerebral hemispheres despite the fact that most of the patients made a complete recovery (47). EEG findings were reported in few patients and showed a non-specific diffuse slow rhythm. Lumbar puncture changes were non-specific, most patients presenting mildly elevated white blood cell counts and CSF proteins with normal CSF glucose levels.

### Treatment of WNV associated opsoclonus-myoclonus syndrome

Although several therapeutic agents were used for the treatment of WNV infection, none was proven to be efficient in a randomized controlled trial. The mainstay of therapy is primarily supportive. Reported therapeutic options that have been tried for WNV encephalitis include: immune γ-globulin (IVIG), plasmapheresis, WNV–specific neutralizing monoclonal antibodies, corticosteroids, ribavirin, interferon α-2b and antisense oligomers (5). The rationale for immunoglobulin administration in WNV neuroinvasive disease is based on pre-clinical studies and individual case reports. However, one phase I/II randomized controlled trial in patients with WNV encephalitis which evaluated immunoglobulin therapy compared with placebo found no significant differences in outcome between groups (48). The lack of efficacy of immunoglobulin treatment might be explained by the fact that human patients already have IgM antibodies at the time of diagnosis and have cleared systemic viremia (49).

Two of the reported patients with WNV associated OMS were treated with IVIG and steroids, one with IVIG and Rituximab and one with plasmapheresis. The rest of the patients received treatment with corticosteroids or only supportive care. There was no significant difference in terms of speed and degree of neurologic improvement or persisting disability between patients.

### Prognosis of WNV associated opsoclonus-myoclonus syndrome

Serious sequelae in WNV infection are usually encountered only in patients with neuroinvasive disease. Fatality rates reach 10–30% in WNV encephalitis, older age and immunocompromised state being both consistently associated with poor outcome (50, 51). Significant improvement in WNV encephalitis survivors is usually seen during the first year after the acute disease but patient—reported clinical sequelae persist in up to 70% of WNV-encephalitis patients even at 5 years after the initial event (52). Most patients complain of persistent fatigue, cognitive deficits, and muscle aches (51).

Data derived from the reported cases of WNV associated OMS suggest that this form of WNV infection has a more benign course compared to the classical WNV encephalitis. Out of the 13 reported cases in the literature till now, one patient died 4 weeks after onset but other 10 showed almost complete neurologic recovery several months after the acute illness. For two patients long term follow up was not available. Although male sex was reported to be associated with more severe illness and death (50), none of the reported male cases of WNV associated OMS died and the clinical course seems to have been similar in women and men.

## Conclusion

West Nile virus infection is probably an underdiagnosed cause of opsoclonus-myoclonus syndrome and this rare clinical entity is probably underrecognized in patients with West Nile virus infection. Although the number of cases of WNV associated opsoclonus-myoclonus syndrome reported in the literature till now is very small, it seems that the disease has a rather benign course and most patients recover almost completely after several months from disease onset. For the moment, there is no robust data to prove the superiority of any type of treatment against supportive care alone. Further studies regarding the susceptibility of different neural areas to West Nile virus infection will probably lead to a better understanding of the pathophysiology of this disease and the required treatment strategies.

## Ethics statement

Written informed consent was obtained from the pacient for the publication of this case report in accordance with the Declaration of Helsinki and all research was conducted following legal and ethical requirements of the University Emergency Hospital Bucharest.

## Author contributions

AE: clinical assessment; RR: clinical assessment, acquisition of data, manuscript drafting; ET: acquisition of data, manuscript drafting; OB and CT: critical revision of the manuscript.

### Conflict of interest statement

The authors declare that the research was conducted in the absence of any commercial or financial relationships that could be construed as a potential conflict of interest.
